# Navigating HIV prevention policy and Islam in Malaysia: contention, compatibility or reconciliation? Findings from in-depth interviews among key stakeholders

**DOI:** 10.1186/s12889-016-3247-y

**Published:** 2016-07-07

**Authors:** Sima Barmania, Syed Mohamed Aljunid

**Affiliations:** United Nations University-International Institute for Global Health PPUKM Complex, Jalan Yaacob Latiff, 56000 Cheras Kuala Lumpur, Malaysia; International Centre for Casemix and Clinical Coding, Faculty of Medicine National University of Malaysia Jalan Yaacob Latiff, Jalan Yaacob Latiff, 56000 Cheras Kuala Lumpur, Malaysia; Department of Health Policy and Management, Faculty of Public Health Kuwait University Kuwait, Kuwait City, Kuwait

**Keywords:** HIV, Prevention, Islam, Malaysia, Muslim, Policy

## Abstract

**Background:**

Malaysia is a multicultural society, predominantly composed of a Muslim majority population, where Islam is influential. Malaysia has a concentrated HIV epidemic amongst high risk groups, such as, Intravenous Drug Users (IVDU), sex workers, transgender women and Men who have sex with Men (MSM). The objective of this study is to understand how Islam shapes HIV prevention strategies in Malaysia by interviewing the three key stakeholder groups identified as being influential, namely the Ministry of Health, Religious leaders and People living with HIV.

**Methods:**

Thirty-Five in depth semi structured interviews were undertaken with religious leaders, Ministry of Health and People living with HIV in the last half of 2013 using purposive sampling. Interviews adhered to a topic guide, were audiotaped, and transcribed verbatim and analyzed using a framework analysis.

**Results:**

Themes including the importance of Islam to health, stakeholder relationships and opinions on HIV prevention emerged. Islam was seen to play a pivotal role in shaping strategies relating to HIV prevention in Malaysia both directly and indirectly. Stakeholders often held different approaches to HIV prevention, which had to be sensitively considered, with some favouring promotion of Islamic principles, whilst others steering towards a more public health centred approach.

**Conclusions:**

The study suggests that Islam indeed plays an important role in shaping health policies and strategies related to HIV prevention in Malaysia. Certainly, stakeholders do hold differing viewpoints, such as stances of what constitutes the right approach to HIV prevention. However there are also areas of broad consensus, such as the importance in Islamic tradition to prevent harm and disease, which can be crafted into existing and future HIV prevention strategies in Malaysia, as well as the wider Muslim world.

## Background

Malaysia is a multicultural society predominantly composed of a Muslim majority population where Islam is influential in moulding policies, including those that relate to health, sexuality and HIV [1]. Malaysia has a concentrated HIV epidemic amongst high risk groups, such as Intravenous Drug Users (IVDU), sex workers, transgender women and Men who have sex with Men (MSM), with a shift to sexual transmission predominating [2]. The demography of HIV in Malaysia is akin to other countries documented in the South East Asian region, as well as those in the Muslim World, such as the Middle East and North Africa, which have concentrated epidemics in high risk groups such as amongst MSM [3]. Amongst MSM, there has been compelling evidence of the need to focus on this key group [4], where often social, cultural and other structures render this group vulnerable to acquiring HIV. In Asia the prevalence of HIV amongst MSM is rising often associated with practices such as ‘chem’ sex, a term used to describe sexual activity under the influence of drugs [5].

In Malaysia, despite the rate of HIV transmission from male to male sex being 19 %, only 0.2 % of the total HIV prevention budget was allocated to MSM programming [6]. Certain biological factors such as unprotected receptive anal intercourse with internal ejaculation within the MSM community [7], as well as religious and cultural factors may mean that MSM are less likely to be involved in prevention programmes for fear of being found out as being gay.

Sex workers are also vulnerable to HIV in Malaysia with increased prevalence of HIV among this group [8] within a context where sex work is illegal. Transgender women have a higher risk of acquiring HIV globally, participate in high risk receptive anal sex and are placed at a high burden of risk including stigma and discrimination in health care settings as a possible hindrance for accessing prevention and treatment [9]. In Malaysia, the prevalence of HIV among transgender women is 5.6 %, although this could be an underestimation [10], with recent documentations of human rights abuse within the community [11]. Women are more vulnerable to acquiring HIV due to cultural, social and economic factors, leaving little room to negotiate safe sexual practices. In addition, due to the strong forbiddance of same-sex relations in Islam, men who have sex with men in Malaysia face social pressures and may often have a wife or girlfriend with whom they have sexual relations.

Malaysia has made great progress in terms of harm reduction, an approach used within the context of HIV prevention amongst IVDU, by implementing needle exchange and has shown great leadership in this area [12]. However, this too had to be carefully navigated to garner support from the imams, utilising principles of the ‘lesser and greater harm’ in the face of rising HIV levels, which now has become commonplace. Notwithstanding, although great strides have been made in harm reduction amongst IVDU, utilising Islamic principles of preservation of life, the same cannot be said of prevention of HIV attributed through sexual transmission [13], which has been significantly underfunded in comparison [14]. There has been considerable Islamic engagement in Malaysia with respect to tackling HIV, both in terms of sensitizing imams to HIV, utilizing Islamic principles as well as religious leader involvement in instigating premarital HIV tests for Muslim couples. However promotion of other HIV prevention measures, such as condom use and cultivating an enabling environment is much more difficult given that many of the activities of high risk groups vulnerable to HIV are forbidden in Islam and thus difficult to reconcile with the norm of current HIV prevention strategies in the non-Muslim world. While there is ever increasing interest in how culture affects health [15], the same cannot be said of religion [16]. Although there is substantial information and research relating to HIV in Malaysia, there is very little relating to HIV in Malaysia and Islam, with a gap of knowledge relating to how Islam affects HIV prevention policies in Malaysia. There is a pressing need to critically analyse how perceptions of Islam actually affect HIV prevention policy in Malaysia, from a neutral, public health perspective, cognisant of and sensitive to the Islamic and political context. Thus, the main objective of the study is to understand how Islam actually and practically shapes HIV prevention strategies in Malaysia. This study contributes to understanding regarding HIV amongst Muslims in Malaysia and the wider Muslim World, with a view to making concrete recommendations for policy and practice.

## Methods

The study described in this paper constitutes part of a larger study which includes both qualitative and quantitative methods which comprised distribution of surveys assessing stakeholders’ knowledge about HIV. However, this paper describes only the findings of the qualitative methods component.

A literature review was undertaken using OVID, PUBMED and PROQUEST including search terms ‘Islam/Islamic/Muslim’, ‘faith’, ‘HIV/AIDS’ and ‘Malaysia’ with no limits given the limited results yielded. In addition, ‘grey literature’ was obtained via the internet which included conference proceedings, organisational reports and UN documents after which a key understanding of the myriad of stakeholders involved in HIV prevention in Malaysia became apparent.

Participants from the three key stakeholder groups involved in HIV prevention policy, namely the Ministry of Health officials, Religious leaders and People Living with HIV within the Klang Valley area (Kuala Lumpur and Putrajaya), were recruited from June 2013 to December 2013 after ethical approval was obtained from UKM Medical Centre. 35 semi-structured in depth interviews were conducted on participants from the three major stakeholders identified as playing a role in HIV prevention policy and strategy; Ministry of Health officials, religious leaders and people living with HIV. The breakdown of the stakeholders interviewed comprised of 11 from the religious leader group, five from the Ministry of Health group, after which saturation was reached, and 19 of the group representing people living with HIV. Participants were recruited using purposive sampling techniques and people living with HIV comprised, Men who have Sex with Men (MSM), Transgender women (TG), Sex Workers (SW) and heterosexual women, recruited through a local Malaysian NGO. Included in the PLHIV group, were four Transgender women, five Men who have sex with Men and four heterosexual women.

Inclusion criteria necessitated that participants were Muslim and over 18 years of age and participants were fully briefed to understand the objectives of the study. Interviews were conducted by the same individual, were face-to-face, audiotaped using a digital recorder and conducted using a topic guide, which included participant’s views on HIV, health in general and the importance of Islam, with the process creating standardization. Further questions explored participants’ knowledge of HIV prevention and policies in Malaysia, as well as perceptions of sex outside marriage, men who have sex with men and condom usage. Interviews were 60 to 90 min in duration, informed consent was obtained both verbally and in written form and on occasion in-house translation was used. Every opportunity was made to ensure participants felt comfortable, understood the purpose of the study and to reassure them of confidentiality. Great care and attention was made to ensure that the interviewer was dressed in religiously appropriate attire when attending meetings with any religious leaders, this included the covering of head and limbs in loosely fitting material respecting norms and local custom. Interviews were transcribed verbatim by a professional transcription company while notes were taken throughout the interview, to document non-verbal communication. The analysis of the data was undertaken in a detailed and systematic way; as soon as the data was collected the recording of the interview was listened to again to understand and familiarize the researcher with the data, often with additional notes being taken. After the transcriber had produced a copy of the interview, in verbatim form the word document was checked and compared with the audio file recording to ensure the transcription was thorough, correct and had no mistakes. Each transcript was printed and reread in detail, on a line by line basis, and then reread again this time with annotations and emerging themes noted. After about a third of the full sample of 35 had been read, which comprised all three stakeholders, a framework analysis was designed which included themes and subthemes. This framework was used to read and analyse through all the interview transcripts from scratch and provided a method of categorizing themes. These themes included Islam’s view of life, health and wellbeing, knowledge about HIV in general, sex outside marriage and current HIV prevention practices in Malaysia, such as condom distribution. In addition, themes such as stakeholder relationships, action to be taken and issues around transgender women and men who have sex with men were identified through the analysis.

## Results

From the participant interviews ten central themes were generated in total, these were as follows: View of life and health in Islam, Sex outside marriage, Understandings of HIV, HIV prevention in Malaysia, Use of Condoms, Transgender women, Men who have sex with Men, Law and Authority, Stakeholder relationships, and Action to be taken.

Furthermore, there were also a number of overriding themes such as the use of language in the context of HIV discourses.

### View of life and health in Islam

The responses generated from the participants in the interviews described various themes such as their views of the place of Islam within their lives. The participants considered Islam as a religion which was all inclusive, pertaining to matters of this world and the next, thus health was seen in a holistic way, not just confined to the body, but mind, soul and the collective health of the Muslim community as a whole.

This viewpoint is demonstrated clearly in the quote below, from someone within the PLHIV MSM community:

*“[Islam]…is a way of life, how do you live every day, what you always do and it’s a relationship between you and God” (PLHIV)*

Islam was seen by participants as a highly practical religion, governing their life, health and activities, illustrated in the response below:

*“…. There are so many guidance given in Islam, either through the Qur’an or the prophetic traditions on how Muslims should look after themselves in terms of health, in terms of diet, in terms of keeping one healthy…” (Religious leader)*

This Islamic guidance, included and influenced attitudes to sexual activities and intimate life.

### Sex outside marriage

*“Ok, to me, as far as I am concerned, any sexual relationship outside the confines of marriage. Haram (forbidden). Meaning, sexual relationship should only be confined to legal marriage between a man and a wife. Man and woman, sorry. Outside that, it’s haram” (Religious Leader)*

The response above, from a religious leader, is typical of those participants interviewed who regard sexual activity outside the confines of marriage as wholly forbidden in Islam.

However, there was also, an acknowledgement amongst many interviewed, that sexual activity outside the boundary of marriage, amongst Muslims in Malaysia occurred; often seen as a corollary of Western influence.

*“[Sex] without marriage it’s haram. You cannot but here it’s natural, especially for teenagers. Have a feeling to try, we can’t stop.....” (PLHIV)*

Although there was a clear understanding amongst the participants of the Islamic ideology that prohibits sex outside marriage, there was often a realisation that endeavouring to stop such activities would be futile.

### Understandings of HIV

All the participants interviewed had some awareness of HIV but expressed that there was not enough openness about the disease because it was associated with negative activities, such as drug use and sexual activity, which is forbidden in Islam.

*“I think Islam is not too… open about this disease, because when we talk about HIV, in Islam we talk about.... we do free sex… and a bad thing” (PLHIV MSM)*

Thus, those people living with HIV often had to interpret their diagnosis, within the cultural and religious framework of Islam, illustrated below succinctly by a participant.

*“Oh I am positive, should I treat this as a sin being given by God or should I treat this as a reminder for me to have a good health?” (PLHIV MSM)*

### HIV prevention in Malaysia

HIV prevention in Malaysia was deemed on the whole relatively positively, participants commending the needle exchange programme and pre-marital HIV screening tests. However there was a sentiment amongst some stakeholders that much more could be done to reduce HIV transmission, such as promotion of condom usage.

*“…just to do simple practical public health things…it’s very, very hard in this country” (PLHIV)*

Or at least, simply undertaking basic public health measures to prevent HIV were obstructed by Islam in some way.

*“…I think there is a long way to prevention in HIV AIDS in Malaysia and mostly for the Muslim communities. Because we cannot help because their religious obligations and their health obligations; it’s a different thing…” (PLHIV)*

### Use of condoms

The issue of condom usage was contentious; the typical response was that condoms were seen in a negative light and signified something immoral as it related to extra marital sexual activity and participants expressed that there were certain societal opinions of condoms.

“They are still taboo; condoms some can accept it, some people don’t…” *(PLHIV TG)*

While religious leaders, in general did not agree with condom usage amongst those out of wedlock. Condom use was permitted in sero-disconcordant couples under the doctrine of preventing harm, with Islamic ideology used to enable actors to reframe the problem, from a moral one to an infectious disease model, articulated in the quote below from an official from the Ministry of Health:

*“Disease prevention, as under disease prevention....so we don’t go into the moral aspects of it as health workers” (MOH official)*

### Transgender Women

Transgenderism, or changing one’s gender was simply not recognised by religious leaders across the spectrum, with only two genders existing in Islam, male, female, or in rare cases ‘Khunsa’ (indeterminate sex). Participants expressed that transgender women were often marginalised, stigmatised and associated with sex work.

*“They perceive a transgender as not a healthy community and transgender is a community that will ….link to sexual activities like sex” (PLHIV MSM)*

Furthermore, some participants believed that due to the Islamic perception of transgender women, the group were driven underground and harder to reach.

*“It poses huge challenges for HIV prevention work because of the stigma and discrimination transgenders are driven underground and are afraid to come forward to access prevention and care services from mainstream providers” (PLHIV)*

### Men who have sex with Men

Participants were clear that homosexuality and men having sexual activity with men was forbidden in Islam but existed in Malay society. As a result MSM were considered to be a hard to reach group to access to provide HIV prevention services to.

*“Homosexuality I think yeah, from the experience which I have at ground level, they are very…very…very hard to reach group” (MOH official)*

Furthermore there was a sentiment by many participants that there were insufficient measures put in place to address prevention amongst MSM and this was particularly evident in the lack of financial resources committed to facilitate the group.

*“The government doesn’t give funding, especially like for example, in our community for MSM, they don’t tend to give funding to us” (PLHIV)*

### Law and authority

The situation in Malaysia is complicated further by the presence of a dual legal system, penal law and Islamic law (Shariah), which does not provide the sort of legal system and enabling environment which is considered best practice according to UN guidelines.

*“…People keep asking why can’t the government change the law; it’s not an easy process” (MOH Officer)*

While some stakeholders view the legal system, which considers it a criminal offence to engage in sex work, cross dressing and men engaging in sex with men, does hinder access to services and HIV prevention; the Ministry of Health deems that provision of services are not impeded and that such laws are rarely enforced in a meaningful way.

### Stakeholder relationships

Due to the points raised already, there exist significant tensions amongst differing stakeholder groups. For example, while there is collaboration with those living with HIV and from the NGO community with both MOH and religious leaders, there is general consensus that religious leader’s engagement serves to convert them.

*“Ustaad [religious leader]. They said LGBT [lesbian, gay, bisexual, transgender] they are illness; they are mental illness…..’…’ For me to be successful, we cannot involve with them …’…’They won’t do anything for us. They still say Haram....they still say Haram” (PLHIV)*

There is distrust amongst the transgender community and religious leaders, as well as with the MOH. Further tensions arise due to funding issues and the rationale of resources, especially with respect to groups that work with MSM communities. Tensions arise due to uncertainty over who holds power and influence, in terms of formulating policy, as illustrated with premarital HIV screening test for married couples. However, further tensions are created as the general population hold a silent influence in their ability to lobby the government.

*“When you tell the general population…oh you are wasting money giving needles….that kind of mind set can happen to them it’s really distract to our programme also”….’…’ sometimes the issue is raised in parliament…you know they raise these kind of issues MAC (Malaysian Aids Council) is giving needles” (PLHIV)*

Whilst further discontent is caused by the perception that the government is being dismissive of both the advice, and financial needs of some NGOs, especially in marginal groups, such as MSM.

*“….as an NGO, you were to tell the Government, the Government wouldn’t event look at you, wouldn’t even listen to you, but for example you have UN agencies doing it, they take a different view of it …you know, they will listen” (PLHIV)*

### Actions to be taken

All stakeholders are committed to preventing HIV, but have differing stances on how this is best achieved, with religious leaders believing that the best way of preventing HIV is to return to Islamic teaching, relating to sexual activity.

*“Abstinence is the best, ok… They have to avoid Zinah [illicit sexual activity]. Whatever becomes obstacles in front, abstinence is still the best. So the education to give the condoms, or other forms is not ok…we have to give the awareness programme, with the Islamic provisions” (Religious leader)*

However, those living with HIV and Non-governmental organisations deem it necessary for a more practical, less moralistic harm reduction strategy to be incorporated.

*“.... actually we have free condom campaign but our…our people in Malaysia can’t accept the campaign because…they say we are Malaysian countries, Muslim countries…” (PLHIV)*

In the middle of these two poles, reside the stance of the Ministry of Health who are in the difficult position of trying to deal with a concentrated HIV epidemic as public health physicians.

*“We are rather quiet about the behaviour but personally, I can say that…as long as we are Muslim, we don’t agree with this behaviour. But we are trying to be professional in our work here…we are looking at the transmission part…we are wearing different hats” (MOH Officer).*

Whilst simultaneously having to reconcile the personal aspect of navigating practices that are essentially considered un-Islamic, are uncomfortable with, but often feel they are actively participate in that sin by association ‘Suhaba’.

*“There is no sex outside marriage. So if anybody wants to say that how condom is permissible, Qur’an quote permissible outside marriage in Islam, I find that hard to swallow because as a principle, there is no sex outside marriage in Islam, so how can you bend something, bend by words…in words or whatever…how can we say something is…you know, how can you want to prevent something which is not right?” (MOH official)*

## Discussion

Those in service delivery and policy makers have on occasion differing viewpoints, for example, the stakeholders from the religious group fundamentally believe that the only long term way of reducing the prevalence of HIV is by, “going back to Islam” an expression heard frequently. This includes the promotion of abstinence of sexual activity until marriage and most certainly does not condone or endorse safer sexual practices outside of marriage, such as condom distribution as this is in opposition to their central belief system.

Within the Ministry of Health, a different viewpoint is taken that endeavours to be more public health focussed, however it must be taken into consideration that they themselves hold their own Islamic perspectives and are subject to being questioned by other groups, including religious leaders and individual tax paying Malaysian Muslims.

Islam affects not just health policy and power but also the process and the manner in which policies are implemented and this is apparent in the method that the premarital HIV screening test came to existence, which is still necessary for Muslim married couples and not non-Muslims. There are certain tactics utilized to simply and crudely ‘get things done’ as expressed by a number of participants. This either meant that activities of certain programmes, such as within the NGO community advocating for marginal groups such as preventing HIV amongst men who have sex with men had either to be done “silently” or at least not to overtly advertise their work, otherwise it can jeopardise other stakeholders and even funding.

In such cases, it is observed how important language is and how it is used to neutralise and placate stakeholders, by focussing on health rather than rights, and emphasis of the term favoured by the Ministry of Health, “prevention of disease”. Some of the language in use highlights existing stakeholder tensions, stigma and discrimination and implies moral and religious judgement such as expressions like ‘prostitution’ or ‘sodomy’ and steering away from such words to alternatives such as ‘sex worker’ and anal sex may be needed to shift focus and change the paradigm to a more public health centred one.

It is also worth mentioning that although Malaysia’s predominant population consists of Muslims, many who are living with HIV, or at risk of HIV infection may not be Muslim; yet, they also are influenced by Islamic religious beliefs on HIV prevention policies and one can ask whether their needs are met. However, perhaps this discrepancy could be seen as an asset and a way of circumventing the rules, by reiterating the statement that it is not just Muslims who are affected by HIV but non-Muslims of other religious denominations and that as part of Malaysia being a multicultural, multi-ethnic and multi-religious society the government and health policies that include Non-Muslims must be catered for too.

In many ways, the issues described are not exclusive to Malaysia, but many Muslim countries which are also forced to formulate strategies to cope with an increase in the cases of HIV. Many predominantly Muslim majority countries such as Iran [17] and the Middle East and North Africa region [18] have had to acknowledge concentrated HIV epidemics which are confined to high risk groups, such as men who have sex with men, and IVDU in the case of Iran. Hasnain [19], in her analysis of approaches to HIV harm reduction describes how it is often common practice of policy makers in Muslim countries to respond with a “propagation of Muslim ideals”, namely avoidance of sexual practices outside the confines of marriage, the primary stance of Islamic religious bodies in Malaysia.

### Limitations

Firstly, there was difficulty in gaining access to the participants to be interviewed due to the sensitivity of the research topic. This issue was addressed by obtaining approval from the stakeholders and NGO involvement to recruit possible participants for interviews from within the high risk groups.

Secondly, there was at times a language barrier as even though participants were chosen who were fluent in English, participants often spoke impromptu in Malay, or felt more comfortable speaking in Malay. This was addressed by using in- house translation, but ideally a professional translator would be used, however in this study, budget constraints limited this option.

Thirdly, the interviews were conducted within one location, the Klang Valley, an urban area which may differ from rural areas within Malaysia, as such there is an acknowledgement of the need for further research to buttress the findings within a wider context.

## Conclusions

The study suggests that Islam indeed plays an important role in shaping health policies and strategies related to HIV prevention in Malaysia. Certainly, stakeholders do hold differing viewpoints, such as stances of what constitutes the right approach to HIV prevention. However there are also areas of broad consensus, such as the importance in Islamic tradition to prevent harm and disease, which can be crafted into existing and future HIV prevention strategies.

Religious leaders should be commended on some of the work that they have done, such as provision of Islamic burial services for those who have died from AIDS and be utilised as an entry point within communities to disseminate correct information about HIV, perhaps during the Friday Khutbahs (sermon). In addition, religious leaders can play an important role in mitigating the stigma and discrimination faced by those living with HIV, as well as providing information, or referring to NGOs and this should be highlighted.

An emphasis on the Islamic principles of compassion and being merciful in the HIV response as well as reminders of the importance of health and preservation of human life in Islam, ought to be reinforced. Renewed debate amongst stakeholders and specifically with Islamic scholars and religious leaders regarding principles that may, or may not, be appropriate in discussions relating to the HIV response is recommended. Concepts such as *Maslaha*, what is in the public interest, *Isthtihad* and greater and lesser harm (*darar*) are ideas that could be looked more deeply in to.

There needs to be a genuine appreciation and acknowledgment that as much as individuals may endeavour to be neutral and impartial no one leaves their personal views behind entirely when in a professional capacity and that for some individuals working in HIV in Malaysia may feel conflicted, for example the issue of distributing condoms. In such cases there should be the availability to ‘opt out’ of participating in a certain practice, for example under the General Medical Council guidelines for personal, ethical or religious reasons, as in the case of not providing termination of pregnancy (but referring the patient to someone that does) without being seen to neglect their professional duties. Such measures may be controversial but they do allow an avenue for those medical doctors to not contravene either their professional duty or moral belief. Thus having the option for those within any organisations (for example MOH) to opt out or be transferred to a different department and be replaced with someone who has less of a personal objection (a non-Muslim counterpart perhaps) may provide a better and more comfortable working environment for all stakeholders. There ought to be meaningful dialogue between those stakeholders involved in HIV prevention, including Ministry of Health, religious departments and PLHIV groups including transgender women, MSM and sex worker communities. The dialogue might specifically concentrate on understanding the issues and viewpoint of the other stakeholders to overcome some of the suspicion and distrust between stakeholders, so that all parties are able to work more closely as a team, whilst also feeling comfortable enough to disagree agreeably. A deliberate change in the language used in terms of written documentation, as well as in spoken discussions, formally and informally, between and within stakeholders may facilitate such dialogue.

Most importantly, personal views should not to be imposed but rather the acknowledgement of the existence of differing viewpoints amongst stakeholders and the extent to which these standpoints can be shaped by perceptions of Islam.

Further research is needed, to not only support the findings but also to study and understand the short and long term impact of public health seen within a religious context and more broadly, the possible ethical implications for patients of service providers.

## Abbreviations

IVDU, intra venous drug user; MAC, Malaysian aids council; MOH, Ministry of Health; MSM, Men who have sex with men; NGO, Nongovernmental organisation; PLHIV, people living with HIV; SW, sex worker; TG, transgender
